# Excess Winter Mortality and Cold Temperatures in a Subtropical City, Guangzhou, China

**DOI:** 10.1371/journal.pone.0077150

**Published:** 2013-10-08

**Authors:** Chun-Quan Ou, Yun-Feng Song, Jun Yang, Patsy Yuen-Kwan Chau, Lin Yang, Ping-Yan Chen, Chit-Ming Wong

**Affiliations:** 1 Department of Biostatistics, School of Public Health and Tropical Medicine, Southern Medical University, Guangzhou, Guangdong, China; 2 Intensive Care Unit, Guangdong No.2 Provincial People’s Hospital, Guangzhou, Guangdong, China; 3 Department of Community Medicine, School of Public Health, The University of Hong Kong, Hong Kong, Hong Kong; 4 Division of Health System, Policy and Management, The Jockey Club School of Public Health and Primary Care, The Chinese University of Hong Kong, Hong Kong, Hong Kong; The Ohio State University, United States of America

## Abstract

**Background:**

A significant increase in mortality was observed during cold winters in many temperate regions. However, there is a lack of evidence from tropical and subtropical regions, and the influence of ambient temperatures on seasonal variation of mortality was not well documented.

**Methods:**

This study included 213,737 registered deaths from January 2003 to December 2011 in Guangzhou, a subtropical city in Southern China. Excess winter mortality was calculated by the excess percentage of monthly mortality in winters over that of non-winter months. A generalized linear model with a quasi-Poisson distribution was applied to analyze the association between monthly mean temperature and mortality, after controlling for other meteorological measures and air pollution.

**Results:**

The mortality rate in the winter was 26% higher than the average rate in other seasons. On average, there were 1,848 excess winter deaths annually, with around half (52%) from cardiovascular diseases and a quarter (24%) from respiratory diseases. Excess winter mortality was higher in the elderly, females and those with low education level than the young, males and those with high education level, respectively. A much larger winter increase was observed in out-of-hospital mortality compared to in-hospital mortality (45% vs. 17%). We found a significant negative correlation of annual excess winter mortality with average winter temperature (r_s_=-0.738, P=0.037), but not with air pollution levels. A 1 °C decrease in monthly mean temperature was associated with an increase of 1.38% (95%CI:0.34%-2.40%) and 0.88% (95%CI:0.11%-1.64%) in monthly mortality at lags of 0-1 month, respectively.

**Conclusion:**

Similar to temperate regions, a subtropical city Guangzhou showed a clear seasonal pattern in mortality, with a sharper spike in winter. Our results highlight the role of cold temperature on the winter mortality even in warm climate. Precautionary measures should be strengthened to mitigate cold-related mortality for people living in warm climate.

## Introduction

Despite of increasing concerns about public health effects of global warming, there is a recurring phenomenon that overall mortality rate is much higher in winter than in summer. Marked excess winter mortality has been observed in many cold regions, although the timing and magnitude of this seasonal peak varied from region to region [1-5]. Recent studies have increased the awareness of excess winter mortality under warm climate. An analysis on the data from 24 countries reported higher increases in coronary event rates during the cold periods in warm regions compared to cold regions [6]. A survey among eight European countries [7] also reported that cold-related mortality was greater in warmer regions than in colder regions. In a study of 11 United States cities, greater effects of cold stress on mortality were observed among the Southern than Northern cities [8]. Although these studies suggested that people living in warm regions probably experienced a higher mortality risk due to cold weather than those living in cold regions, excess winter mortality in tropical and subtropical regions still remains unclear, resulting in uncertainties in formulating public health intervention strategies in these regions.

Many factors have been found to determine excess winter mortality, including influenza epidemics, cold stress, individual lifestyle risk factors and socioeconomic factors [2,3,9,10]. Among them, cold temperature is regarded as a major factor contributing to excess winter deaths. Experimental studies have shown that cold temperature causes change in blood pressure, vasoconstriction, and increases in blood viscosity and levels of plasma fibrinogen and cholesterol, thereby triggering thromboembolic events [1,11,12]. Inhalation of cold air can also cause the lung airways to narrow and produce phlegm, leading to an increased risk of bronchitis, pneumonia and acute exacerbation of chronic lung diseases. Nonetheless, even when appropriate, medical examiners do not routinely record these causes of death as cold-related. Cold weather-related deaths are substantially underestimated [1]. A lot of efforts have been put on providing epidemiological evidence on the short-term effects of daily cold temperatures on morbidity and mortality in many regions including mainland China [9,13-20]. However, seasonal variation of mortality and its association with changes in ambient temperatures was not assessable in previous studies based on daily data, but should be based on aggregated monthly data. In this study, we assessed the excess winter mortality and examined its relation to cold temperatures in Guangzhou, a subtropical city in Southern China.

## Materials and Methods

### Data collection

**Figure 1:**
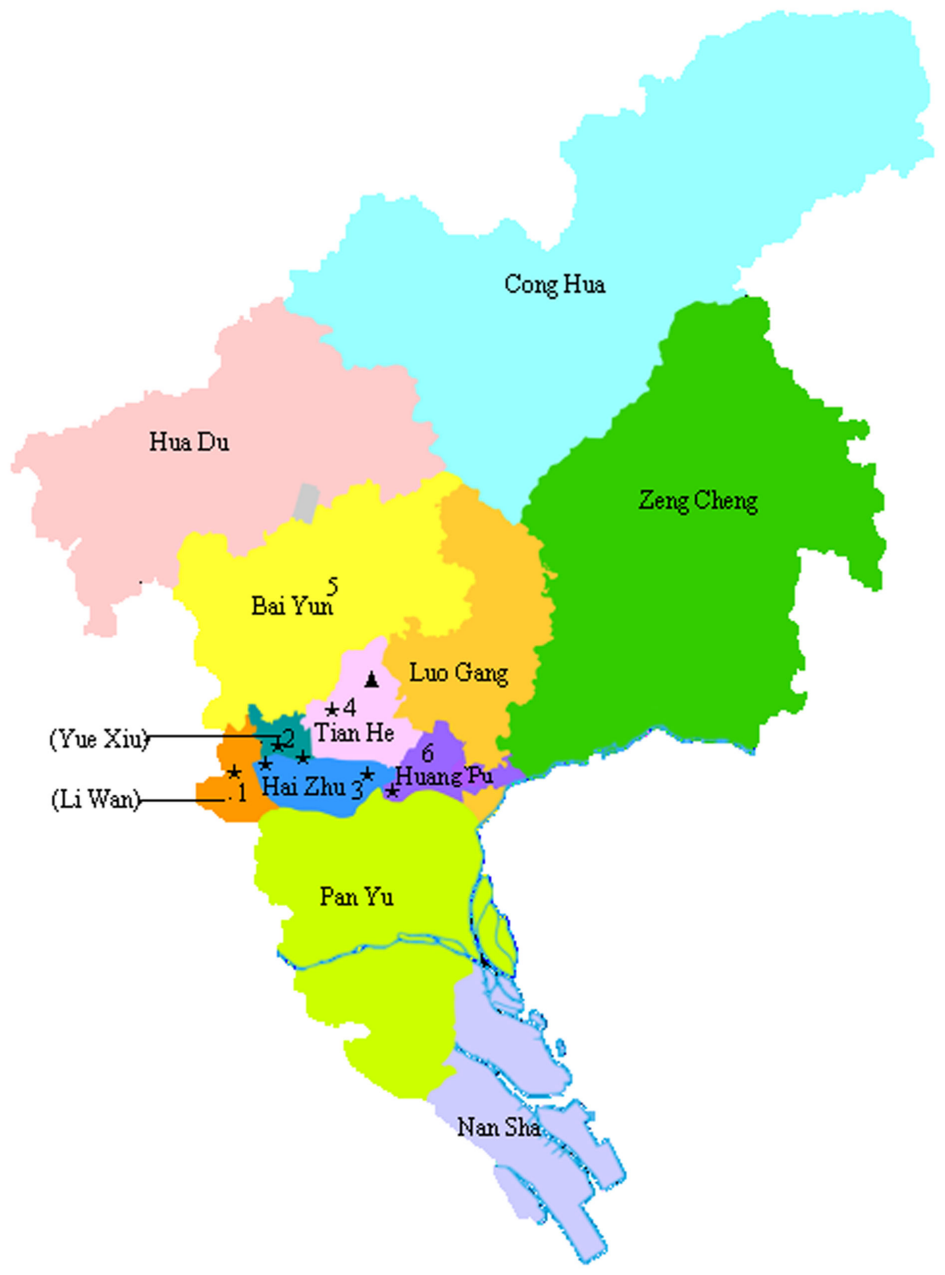
A map of Guangzhou showing the location of Guangzhou weather station (marked by triangles) and seven air pollution monitoring stations (marked by a star). The six urban districts included in the present study are labeled by number 1-6.

Guangzhou is one of the largest metropolitan cities in Southern China. It is located at 23.17°N latitude and 113.14°E longitude (Figure S1). It is 7,434 square kilometer with a population of 12.7 million in 2010 reported by the sixth national population census. 6.62% of the population is the elderly aged over 65, and 42.2% have high school or higher education attainment. The spatial coverage and administrative division of Guangzhou had some changes during the study period from 2003 to 2011. All air pollution monitoring stations and weather station was located at central districts (Figure 1). Considering the consistency of the study population and the validity of data, our study included six central urban districts where there are 7.7 million permanent residents, accounting for 60.8% of Guangzhou population in 2010. We collected individual data from the Guangzhou Center for Disease Control and Prevention, for all registered deaths in these six urban areas between 1 January 2003 and 31 December 2011. These data included cause of death, date of birth, date of death, sex and education level. Causes of death were classified by the Tenth Revision of the International Classification of Diseases (ICD-10). We examined all-cause mortality and mortality due to cardiovascular diseases (I00 - I99), respiratory diseases (J00-J99), stroke (I60 – I69), ischemic heart diseases (IHD, I20 - I25) and chronic obstructive pulmonary diseases (COPD, J40-47).

We obtained daily meteorological data in Guangzhou from the National Meteorological Information Center in China (http://www.nmic.gov.cn/web/channel-464.htm). These meteorological data were collected from Guangzhou Weather Station, which is located at 23.10°N latitude and 113.20°E longitude in the urban area of Guangzhou. This station is the only basic weather station in Guangzhou that is part of the global meteorological information sharing network, and has been providing metrological data in Guangzhou since 1951.

We obtained air pollution data for seven monitoring stations on daily concentration of particulate matter less than 10 µm in aerodynamic diameter (PM_10_), nitrogen dioxide (NO_2_) and sulfur dioxide (SO_2_) from Guangzhou Bureau of Environmental Protection. The average concentration for these seven stations was calculated.

### Data analysis

We standardized all months to have 30 days to calculate monthly mortality [3,21]. That is, the standardized monthly number of deaths is calculated by daily average deaths multiplied by 30. Excess winter mortality was estimated by the winter: non-winter ratio of average monthly deaths minus one, that is, the percentage that the observed winter deaths is above that which is expected from the non-winter deaths. Firstly, we calculated the average excess winter mortality for the whole study period (Jan 2003-Dec 2011) by comparing average monthly mortality in the winter months (December to March) to that in the rest months (April to November). The average excess winter deaths per year were calculated by the difference in average monthly number of deaths between winter and non-winter multiplied by 4 (i.e. the number of winter months per year). Then, to explore any annual changes in excess winter mortality, we estimated annual excess winter mortality by comparing average monthly mortality between the four winter months with the previous (August-November) and subsequent four months (April-July). Spearman correlation was performed to determine the association between annual excess winter mortality and environmental measures in the winter.

Generalized linear models have been widely used to determine the short-term effect of daily air pollution or ambient temperatures on mortality, which were estimated after adjusting for seasonal effects and long-term trend. The seasonal effect, instead of daily variation, is our interest in the present study. Aggregating daily mortality into monthly mortality, we fitted a polynomial distributed lag model with a quasi-Poisson distribution to determine the influence of monthly mean temperature on mortality at lags of 0, 1 and 2 months simultaneously. The effect was quantified by the percentage change in monthly mortality associated with 1°C increase in monthly mean temperature. We also estimated the potential extra effect of frequency of extreme temperatures by including monthly number of extremely hot days and number of extremely cold days in the model. Extremely cold or extremely hot days were defined as the days with the mean temperature lower than the 5th percentile or higher than the 95th percentile of the temperature distribution, respectively. Monthly mean air pollution and other meteorological parameters (relative humidity, atmospheric pressure, precipitation, sunshine and wind speed) were all controlled for in the model.

All statistical analyses were performed using *R* (The R Foundation for Statistical Computing, version 2.15.2).

### Ethic statement

The Ethics Committee of Southern Medical University where this study was carried out has approved the study proposal. Informed consent was not required because the data used in this study were official death registration records which were analyzed anonymously.

## Results

### Excess winter mortality by subpopulations

**Figure 2:**
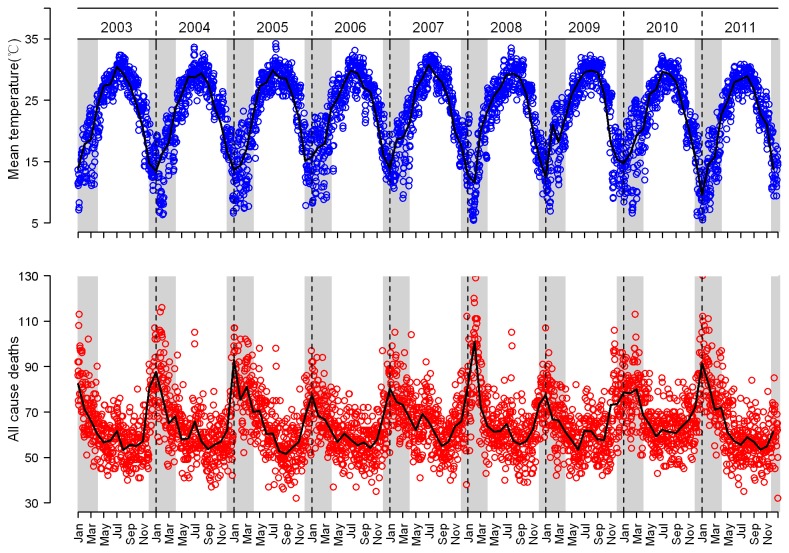
Daily mean temperatures (blue dots) and daily number of all-cause deaths (red dots) in Guangzhou, China. The line represents monthly average and winter months are highlighted in grey.

Descriptive statistics of meteorological measures are shown in Table 1. Ambient temperatures showed a clear seasonal pattern (Figure 2). Warm wintertime temperatures were observed in Guangzhou with an average daily temperature of 15.9°C (range 5.4 to 25.4°C).

**Table 1:** Descriptive statistics of daily temperatures and relative humidity during 2003-2011 in Guangzhou.

						**Percentile**				
	**minimum**	**maximum**		**mean**	**SD**	**5th**	**25th**	**50th**	**75th**	**95th**
**Winter months**										
Mean temperature (°C)	5.4	25.4		15.9	4.4	8.3	12.6	16.0	19.4	22.7
Minimum temperature (°C)	1.8	23.8		12.7	4.6	5.5	9.1	12.6	16.1	20.4
Maximum temperature (°C)	6.2	29.6		20.6	4.9	11.3	17.3	21.4	24.5	27.5
Mean humidity (%)	20.0	99.0		68.8	15.3	39.0	60.0	71.0	80.0	90.0
**Non-winter months**										
Mean temperature (°C)	9.0	34.2		26.1	3.8	18.6	24.1	26.9	28.8	31.1
Minimum temperature(°C)	6.3	30.4		23.0	3.9	15.1	20.8	24.1	25.7	27.7
Maximum temperature(°C)	11.9	39.1		30.6	4.1	23.0	28.3	31.2	33.7	35.8
Mean humidity (%)	26.0	99.0		72.8	11.5	52.0	66.0	74.0	81.0	90.0

For the 9-year period from January 2003 to December 2011, there were a total of 213,737 registered deaths included in this study. During the study period, the mean daily number of all-causes deaths was 65.0, of which 23.8 and 11.8 were from cardiovascular diseases and respiratory diseases, respectively. Figure 2 indicates strongly that seasonal mortality does occur in this subtropical city, Guangzhou. There was a dramatic increase in mortality in winter and a trough in summer, but a slight rise was observed during the hottest period (July to August). An average of 462 more deaths per month occurred in the winter season compared to non-winter seasons. This would equate to an estimated 1,848 excess winter deaths each year, with around half (51.9%) from cardiovascular diseases and a quarter (24.0%) from respiratory diseases. Correspondingly, all-cause mortality in winter was 25.7% higher than in other seasons. The largest excess risk in winter was observed for mortality due to COPD. Regardless of age or socioeconomic status, all subpopulations experienced marked excess winter mortality. Female was more vulnerable than male. The risks increased sharply with age, as people aged over 75 years accounted for 71.6% of excess winter deaths. Those with no formal education or primary education had higher excess winter mortality than those with secondary or higher education. A much larger increase in out-of-hospital mortality compared to in-hospital mortality was observed in winter (Table 2).

**Table 2:** Excess winter mortality by cause, gender, age group and education level in Guangzhou, 2003-2011.

		**Mean monthly deaths**		Annual excess winter deaths (a-b)*4	**Excess winter mortality(%) (a-b)/b**
**Variables**	**Groups**	**Winter (a)**	**Non-winter (b)**		
**Cause**	All	2260	1798	1848	25.7
	Cardiovascular	876	636	960	37.7
	IHD	292	212	320	37.7
	Stroke	298	217	324	37.3
	Respiratory	428	317	444	35.0
	COPD	229	161	272	42.2
	Other*	956	844	448	13.3
**Gender**	Male	1258	1015	972	23.9
	Female	1002	783	876	28.0
**Age(years**)	0-64	518	466	208	11.2
	65-74	471	394	308	19.5
	75+	1270	939	1324	35.3
**Education**	None	413	297	464	39.1
	Primary	937	726	844	29.1
	Secondary or higher	779	669	440	16.4
**Place of death**	In hospitals	954	809	580	17.9
	Out hospitals	673	463	840	45.4

Note: other represents non-cardiorespiratory deaths; IHD: ischemic heart disease; COPD: chronic obstructive pulmonary disease.

### Annual change in excess winter mortality

Annual winter peaks in mortality varied considerably in magnitude. Excess winter mortality ranged from 21.5% in 2006/2007 to 31.4% in 2003/2004 but did not show a clear trend (Table 3). Excess winter mortality was negatively correlated to average temperature in winter with a Spearman’s correlation coefficient of -0.738 (P=0.037) (Table 4). Annual excess winter mortality due to cardiovascular diseases significantly increased with increasing relative humidity in winter (r_s_=0.714, P=0.047). However, annual excess winter mortality was not significantly correlated with atmospheric pressure or the levels of air pollution, including SO_2_, NO_2_ and PM_10_.

**Table 3:** Average temperature in the winter and excess winter mortality by cause and year in Guangzhou.

**Year**	**Temperature(°C)**				**Excess winter mortality(%)**						
	mean	minimum	Extremely Cold days		All	Cardio-vascular	IHD	Stroke	Respiratory	COPD	other
2003/2004	15.6	12.2	19		31.4	44.0	39.0	43.4	41.2	43.8	17.1
2004/2005	15.5	12.5	22		28.5	40.1	43.1	39.9	36.8	43.8	16.2
2005/2006	16.5	13.5	13		24.2	40.3	45.5	35.3	30.0	31.0	11.3
2006/2007	16.8	13.7	3		21.5	32.2	30.8	37.5	15.8	27.4	15.7
2007/2008	15.6	12.4	22		31.1	47.1	53.5	49.6	45.8	59.9	13.8
2008/2009	16.7	13.3	11		22.0	28.3	32.1	25.0	34.4	39.9	13.0
2009/2010	16.4	13.7	21		23.0	29.1	29.9	28.9	33.4	34.5	14.1
2010/2011	13.9	10.5	35		27.2	38.2	36.5	39.0	42.3	45.0	13.1

Note: Extremely cold days were defined as the days with mean temperature lower than the 5th percentile of the temperature distribution over the study period; IHD: ischemic heart disease; COPD: chronic obstructive pulmonary disease; Other represents non-cardiorespiratory deaths.

**Table 4:** Spearman’s correlation between annual excess winter mortality and annual average temperature and levels of air pollution in the winter.

**Environmental factors**	**All-cause**	**Cardio-vascular**	**IHD**	**Stroke**	**Respiratory**	**COPD**	**Other**
Mean temperature	-0.738*	-0.452	-0.429	-0.619	-0.810*	-0.838**	-0.190
Minimum temperature	-0.738*	-0.524	-0.500	-0.667	-0.857**	-0.838**	-0.119
Number of extremely cold days	0.635	0.371	0.359	0.527	0.778*	0.813*	0.084
Relative humidity	-0.571	-0.714*	-0.762*	-0.381	-0.357	-0.311	0.310
Atmospheric pressure	0.095	0.381	0.238	0.476	-0.214	-0.192	0.595
PM_10_	0.095	0.405	0.524	0.262	-0.310	-0.263	0.190
SO_2_	0.381	0.571	0.476	0.357	-0.190	-0.216	0.381
NO_2_	0.381	0.357	0.548	0.119	-0.048	-0.024	0.119

Note: *P<0.05, **P<0.01; IHD: ischemic heart disease; COPD: chronic obstructive pulmonary disease; Other represents non-cardiorespiratory deaths.

### Association between monthly mortality and ambient temperatures

**Figure 3:**
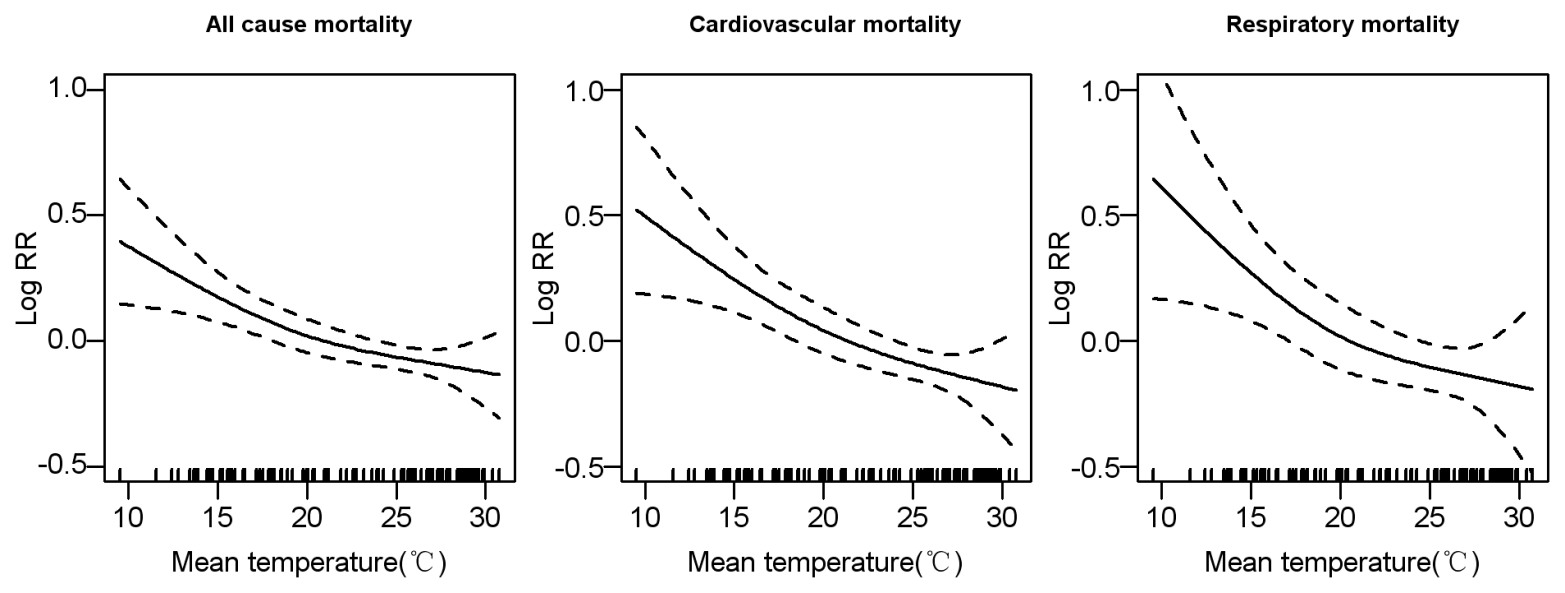
The dose-response relationship between average monthly temperature and monthly mortality using a natural spline function with a degree freedom of 3.

The dose-response curve revealed a negative linear relationship in that a decrease in monthly temperature was associated with an increase in mortality (Figure 3). The goodness of fit indicator R^2^ was 0.807 for the all-cause mortality model. Partial autocorrelation function of the residuals did not appear any significant autocorrelations or any discernible patterns. We observed a 1.38% (95%CI: 0.34%-2.40%) increase in all-cause mortality per 1°C fall in monthly mean temperature and also a significant one-month lagged effect with a corresponding effect estimate of 0.88% (95%CI:0.11%-1.64%). The impact was more remarkable for mortality due to cardiovascular diseases and respiratory diseases, with the greatest effect estimates observed in the same month and at lagged one month, respectively. Stratified analyses revealed that temperature-related mortality had a similar pattern with excess winter mortality, that is, the effect of monthly temperature on mortality was higher in the elderly and those with low education level than the young and those with high education level, respectively. Furthermore, we observed that extreme temperatures had extra effects on mortality. Number of extremely hot days and number of extremely cold days in a month were both positively associated with monthly mortality, and the effect sizes were similar (Table 5). No significant effects on monthly mortality were detected for monthly mean humidity, sunshine, precipitation, SO_2_ or NO_2_, while a significant effect was observed for wind speed and PM_10_ (Table S1).

**Table 5:** The effect of average monthly temperature and frequency of days with extremely temperatures on monthly mortality.

**Variables**	**Groups**	**Mean temperature**			**Number of**	**Number of**
		**Lag0**	**Lag1**	**Lag2**	**extremely cold days**	**extremely hot days**
**Cause**	All-cause	-1.38 (-2.40, -0.34)	-0.88 (-1.64, -0.11)	-0.29 (-0.89, 0.31)	1.07 (0.48, 1.65)	0.93 (0.39, 1.48)
	Cardiovascular	-1.88(-3.19,-0.56)	-1.09 (-2.06, -0.11)	-0.36 (-1.13, 0.41)	1.24 (0.50, 1.97)	1.24 (0.53, 1.95)
	CHD	-1.57 (-3.24, 0.14)	-1.00 (-2.24, 0.25)	-0.48 (-1.46, 0.51)	1.70 (0.78, 2.63)	1.50 (0.60, 2.40)
	Stroke	-2.06 (-3.45, -0.66)	-1.14 (-2.17, -0.10)	-0.05 (-0.86, 0.78)	0.64 (-0.14, 1.42)	0.60 (-0.16, 1.37)
	Respiratory	-1.27 (-3.17, 0.67)	-2.12 (-3.49, -0.73)	-0.28 (-1.38, 0.84)	1.23 (0.16, 2.29)	1.61 (0.61, 2.62)
	COPD	-1.36 (-3.50, 0.83)	-1.96 (-3.54, -0.36)	-0.62 (-1.87, 0.65)	1.43 (0.21, 2.65)	1.61 (0.46, 2.76)
**Gender**	Male	-1.59 (-2.57, -0.60)	-0.68 (-1.41, 0.06)	-0.22 (-0.80, 0.36)	0.98 (0.42, 1.54)	0.69 (0.17, 1.22)
	Female	-1.12 (-2.34, 0.12)	-1.13 (-2.04, -0.22)	-0.38 (-1.10, 0.34)	1.17 (0.48, 1.87)	1.24 (0.59, 1.88)
**Age(years)**	<65	-1.19 (-2.12, -0.25)	-0.16 (-0.87, 0.55)	-0.14 (-0.69, 0.42)	0.55 (0.01, 1.09)	0.38 (-0.11, 0.87)
	65-74	-1.36 (-2.49, -0.21)	-0.52 (-1.39, 0.35)	-0.33 (-1.00, 0.35)	0.95 (0.29, 1.61)	0.59 (-0.01, 1.19)
	75+	-1.50 (-2.83, -0.16)	-1.33 (-2.29, -0.35)	-0.36 (-1.13, 0.41)	1.27 (0.53, 2.01)	1.35 (0.65, 2.06)
**Education**	None	-1.64 (-3.28, 0.02)	-2.19 (-3.39, -0.98)	-0.08 (-1.04, 0.89)	0.94 (0.01, 1.87)	1.63 (0.75, 2.50)
	Primary	-1.93 (-3.22, -0.63)	-1.18 (-2.13, -0.21)	-0.12 (-0.88, 0.64)	1.20 (0.47, 1.94)	1.05 (0.36, 1.74)
	Secondary or higher	-0.57 (-1.62, 0.50)	-0.39 (-1.17, 0.39)	-0.62 (-1.23, -0.01)	1.29 (0.69, 1.89)	0.21 (-0.35, 0.76)
**Place of death**	In-hospital	-2.35 (-9.52, 5.39)	-0.82 (-6.73, 5.45)	1.75 (-2.83, 6.54)	1.60 (-6.18, 2.98)	2.58 (-1.42, 6.58)
	Out-hospital	-3.08 (-7.09, 1.10)	-1.31 (-4.38, 1.85)	0.10 (-2.30, 2.57)	0.90 (-1.43, 3.24)	1.04 (-1.18, 3.26)

## Discussion

The winter peaks of mortality have been intensively investigated in Europe and many other temperate regions [1,4,22], and a few studies have explored mortality peaks in the hot, dry season [23]. The magnitude of cold effect estimates varied by location and study population, and this phenomenon was observed even in coordinated analyses of multiple temperate cities. The multi-city studies in Europe [7], the US [8] and other regions [6] have consistently shown a greater relative risk of mortality associated with cold exposure in regions with relatively mild winter climates. The dose-response curves for the mortality effects revealed a higher temperature threshold in the warm cities than the cold ones [24]. In the present study, we observed a marked seasonal variation of mortality with a peak in months with low temperatures which was also observed in another subtropical city Nairobi [20]. Approximately half of the excess winter deaths were from cardiovascular diseases and a quarter from respiratory diseases, similar with the literature review by Keating [5]. Despite of relatively warm winter in Guangzhou compared to temperate regions, excess winter mortality (25.7%) was greater than those reported in France (15%) [21] and in the United Kingdom (18%) [25]. There are several possible explanations for these findings. People living in warm climate usually poorly adapted to cold exposure in the physiological, social and behavioral aspects [7,26-29].

The determinants of excess winter mortality are not fully understood, but cold weather is usually regarded as playing a dominant role [1,30]. Other factors, such as influenza epidemic, lack of physical exercise [31], lower intake of fresh fruits and vegetables and higher intake of saturated fat during winter [11,32,33], may be also involved in driving the winter mortality peaks. In the present study, we considered a variety of environmental factors and found that the seasonality of mortality was mainly attributable to ambient temperatures.

Although many studies have established a link between weather conditions and mortality on a daily basis [8,16,18,20], little is known about this relationship at a monthly time scale. In contrast to a U- or V-shaped relationship between daily mortality and daily temperature previously observed by us [16] and other authors [8,18,20], this study demonstrated there was a monotonically decreasing relationship on a monthly-basis analysis as also observed in a previous study [34]. This suggests that the effects of temperature on mortality are scale-dependent. A scale-specific effect of weather has been shown on animal behavior [35]. The association at a broader time scale may include some lagged and harvesting effects at a finer time scale and may represent longer term effect. Analyses of the association at multiple time scales could help to better comprehend causal mechanisms of health effects of weather conditions and climate change. Additionally, we observed a significant effect of the frequency of extremely cold days after adjusting for monthly mean temperature, suggesting that winter mortality might have increased due to more frequent cold episodes, even if mean winter temperature did not change or slightly increased.

Since winter excess deaths are attributable to cold exposure, winter deaths can be partly prevented if people keep warm during the winter months by taking effective precautionary measures. A recent report by the Marmot Review Team in the United Kingdoms concluded that cold homes and insufficient fuel due to poverty are risk factors of excess winter mortality [10]. There is no central heating system in Guangzhou and people are not used to wearing thick clothes during cold weather. Although an average of 2.61 sets of air condition were equipped each household in urban areas in Guangzhou [36], air conditioners installed in Guangzhou residences in general have no function of up-regulating temperatures. In most private residences, indoor temperatures in winter are almost the same with outdoor temperatures. There are heating equipments available in nursing homes or hospitals, therefore in-hospital patients are less affected by ambient low temperatures. This is a possible explanation for the particularly higher winter increase in in-hospital mortality compared to out-of-hospital mortality. Our findings highlight the need to strengthen the awareness of potential dangers of cold weather and to enhance coping capacities to mitigate cold-related mortality for residents living in warm regions. Recently, in mainland China, a wide spread attention has been paid to a drastic debate on whether a central heating system is needed to be constructed in Southern China. Our study provides strong evidence for the necessity of this measure.

Identification of vulnerable subpopulation is the primary stage of a targeted intervention for preventing excess winter deaths. Many previous studies have showed that the elderly are vulnerable to adverse cold-related health outcomes [14]. As a result of aging and existing medical conditions, the elderly may not have the physiological capacity to adequately respond to cold exposure. Our study also confirmed this, as excess winter mortality was three times as high in those aged over 75 as in those aged under 65. In the present study, females experienced a higher excess winter mortality, which is consistent with the finding in New Zealand, Australia and the United Kingdom [3,22,37]. There was no conclusive evidence of socioeconomic gradient in cold weather-related mortality. A few studies in Europe did not find clear evidence that area-based socioeconomic deprivation confers vulnerability to winter deaths [3,38] or cold-related deaths [39]. Country-level per capita expenditure on primary and secondary education was also insignificantly associated with winter mortality [4]. In Kent, UK, the highest excess winter mortality was observed in some of the most affluent areas [40]. However, some studies showed that race, individual education attainment and occupational class modified temperature-related mortality [16,41]. In the present study, we used individual education level as an indicator of socioeconomic status and found greater excess winter mortality in those with less education. The social disparity likely reflects housing conditions and medical conditions associated with poverty, and increased occupational exposure to ambient cold temperatures. We performed further analysis of the association between monthly temperature and mortality, and also identified that the elderly, females and those with no formal education was at particular risk of low temperature-related mortality.

Although global climate change has been observed and predicted, no decline in seasonal mortality was evident over two recent decades in New Zealand [3]. Another investigation in the United States reported that relative effects of cold temperatures on cardiovascular mortality remained constant from 1987 to 2000 [42], while few studies investigated the trend of absolute effects of cold temperatures because estimating cold-related deaths is a complex issue. In this study, we did not found an increasing or decreasing trend in excess winter mortality, but a significant negative correlation was observed between annual excess winter mortality and winter mean temperatures. However, this requires cautious interpretations because we only had nine years of data in the present study. The national assessment climate models projected that the national assessment of winter mortality in the United States will decrease slightly but summer mortality will increase dramatically in the period 2020 to 2050 in the United States [43]. However, this result may not be generalized to subtropical or tropical regions. Residents in the tropics are acclimated to high summer temperatures for a long period of time, and therefore elevated temperature in summer may not have significant impact on hot-related mortality. In tropical regions, whether potential decreases in winter mortality offset summer increases remains uncertain. Further research in tropical or subtropical regions is needed to fully understand the possible health impacts of climate variability and change.

There are several limitations in the present study. Firstly, as in many previous studies [3,22,37], we used a very simple method to estimate winter excess mortality. Secondly, we did not adjust for the effects of influenza in the present study because of unavailability of the data, although several studies have indicated that cold exposure has been associated with an increase in the incidence of influenza and some winter deaths were caused by respiratory infections including influenza. Therefore, residual bias may still exist. Thirdly, we included only three ambient air pollutants (SO_2_, NO_2_ and PM_10_) in the model, but there are still many other air pollutants such as particulate matter greater than 10 µm and ozone which could have caused bias in our estimates. Finally, based on this study with nine years of data, we did not find significant trend in the seasonal variation over time. However, our study period may have been too short to reveal the changes in mortality seasonality which may be related to climate change. Further research using a longer study period or climate change projections are warranted to address this question.

## Conclusions

Mortality in Guangzhou presents a clear seasonality in mortality of dramatic rises during winters. Females, the elderly and those with lower education are at high risk of winter mortality. Our study implies that cold temperature may be one of the major environmental factors associated with excess winter mortality, even in the subtropics or tropics. Cold-related mortality should not be underestimated because of recent focus on heat wave episodes. Precautionary measures should be strengthened for people living in warm climates to cope with cold weather.

## Supporting Information

Figure S1 **The geographic location of Guangzhou (marked by a red triangle).**
(TIF)

Table S1 **The percentage change (95% confidence interval) in monthly mortality associated with 1 unit increase in monthly environmental measures.**
(DOC)
