# Supplementary figures and images for: Excess Winter Mortality and Cold Temperatures in a Subtropical City, Guangzhou, China
Source: PLoS One. 2013 Oct 8;8(10):e77150. doi: 10.1371/journal.pone.0077150 (PMC3792910; doi:10.1371/journal.pone.0077150)

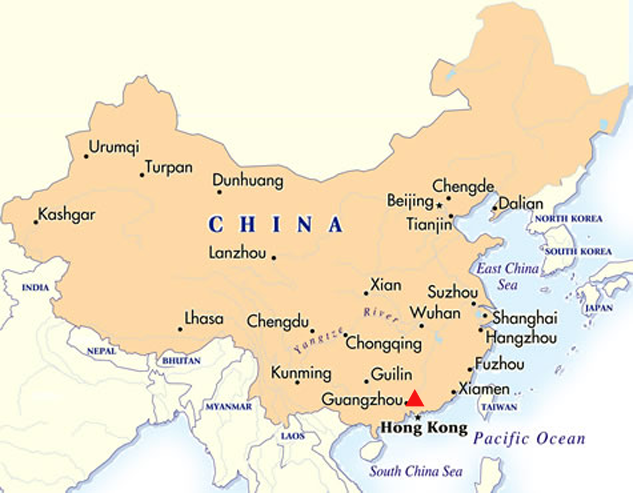

Supplement: Figure S1 — The geographic location of Guangzhou (marked by a red triangle). (TIF) [file pone.0077150.s001.tif]
