# Supplementary material for: Excess Winter Mortality and Cold Temperatures in a Subtropical City, Guangzhou, China
Source: PLoS One. 2013 Oct 8;8(10):e77150. doi: 10.1371/journal.pone.0077150 (PMC3792910; doi:10.1371/journal.pone.0077150)
Supplement: Table S1 — The percentage change (95% confidence interval) in monthly mortality associated with 1 unit increase in monthly environmental measures. (DOC) [file pone.0077150.s002.doc]

**Table S1.** The percentage change (95% confidence interval) in monthly mortality associated with 1 unit increase in monthly environmental measures.

| **Variables** | **All-cause**  **(95%CI)** | **Cardiovascular**  **(95%CI)** | **Respiratory**  **(95%CI)** |
| --- | --- | --- | --- |
| Relative humidity (%) | -0.27(-0.66, 0.12) | -0.58(-1.08, -0.08) | -0.33(-1.06, 0.39) |
| Atmospheric pressure (0.1hPa) | -0.03(-0.12, 0.05) | -0.03(-0.14, 0.08) | -0.04(-0.20, 0.12) |
| Precipitation (0.1mm) | 0.01(-0.01, 0.01) | -0.01(-0.01, 0.01) | 0.01(-0.01, 0.01) |
| Wind speed (0.1m/s) | -0.63(-1.04, -0.22) | -0.93(-1.45, -0.41) | -0.87(-1.62, -0.11) |
| Sunshine (0.1hr) | 0.01(-0.18, 0.18) | -0.10(-0.33, 0.13) | -0.04(-0.38, 0.29) |
| PM_10_ (μg/m^3^) | -0.18(-0.32, -0.03) | -0.25(-0.44, -0.07) | -0.15(-0.41, 0.32) |
| NO_2_ (μg/m^3^) | 0.07(-0.13, 0.28) | 0.09(-0.17, 0.36) | -0.06(-0.44, 0.32) |
| SO_2_ (μg/m^3^) | 0.07(-0.04, 0.17) | 0.11(-0.03, 0.24) | 0.06(-0.13, 0.26) |
